# Red Blood Cell Contribution to Hemostasis

**DOI:** 10.3389/fped.2021.629824

**Published:** 2021-04-01

**Authors:** Andrea H. Gillespie, Allan Doctor

**Affiliations:** ^1^Division of Pediatric Hematology and Oncology, Oregon Health and Sciences University, Portland, OR, United States; ^2^Division of Pediatric Critical Care Medicine, The Center for Blood Oxygen Transport and Hemostasis, University of Maryland School of Medicine, Baltimore, MD, United States

**Keywords:** red blood cell(s), shear rate, aggregation, phosphatidylserine, microparticles, hemostasis

## Abstract

Red Blood Cells (RBCs) have been increasingly recognized to play important roles in hemostasis and the mechanisms by which they do so continue to be elucidated. First and foremost, RBC biomechanics are the principal determinant of viscosity and flow dynamics of blood, which strongly influence all features of hemostasis. Of note, morphologic pathology, such as that found in sickle cell disease, leads to increased risk of thrombotic disease. RBC surface interactions govern signaling between platelets and RBCs and also aid in the conversion of prothrombin to thrombin. Additionally, RBCs generate microparticles which have been shown to reduce clotting time. Finally, blood clot structure and maturation are dependent on the inclusion of RBCs in forming thrombi. Here, we review the above mechanisms of RBC contribution to hemostasis.

## Introduction

**Figure 1:**
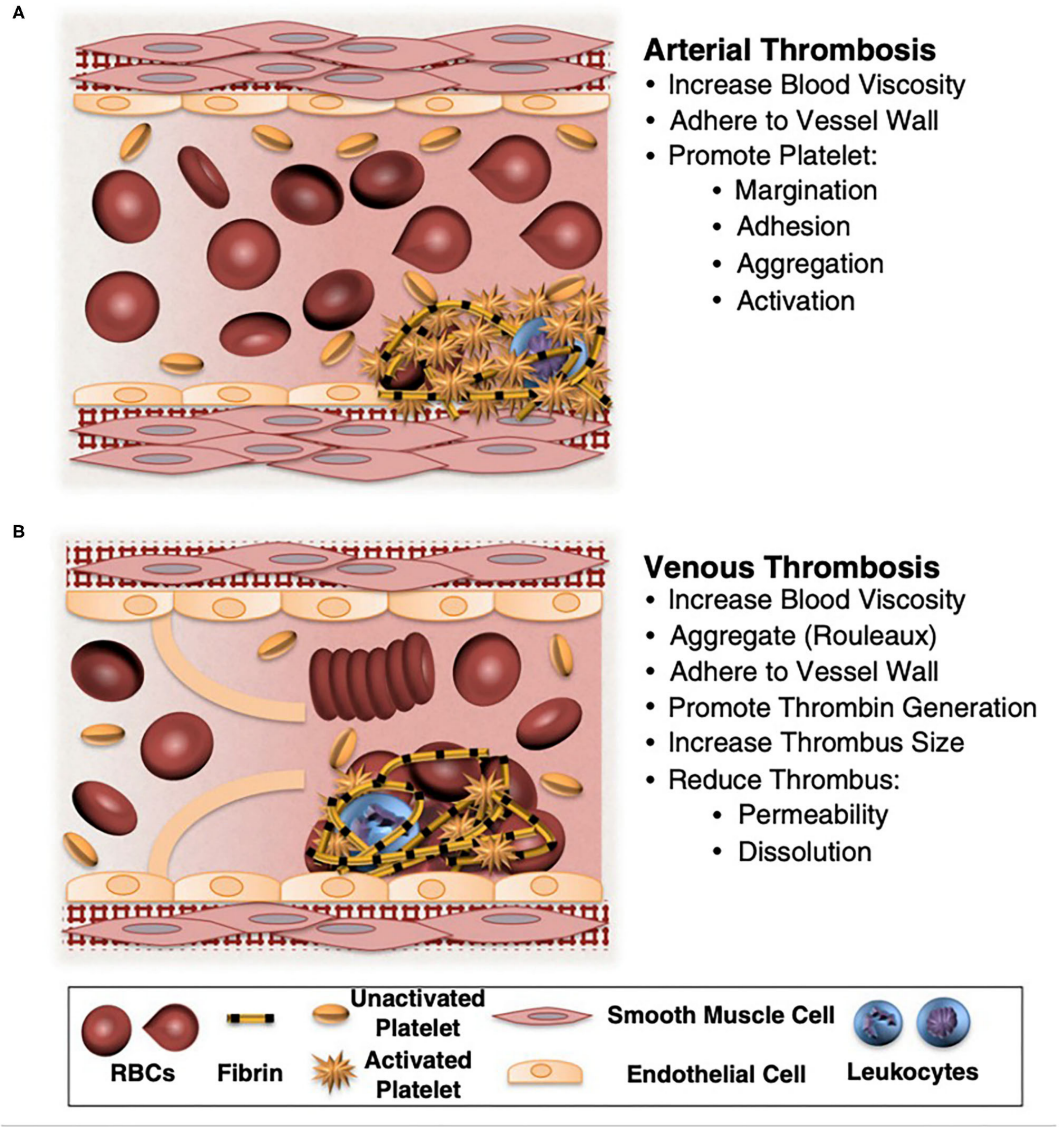
Potential contributions of normal and abnormal RBCs to arterial and venous thrombosis/thromboembolism. **(A)** Arterial thrombi arise in vessels with high shear rates, which promotes the rapid formation of platelet-rich thrombi. During arterial thrombosis, RBCs promote platelet margination, increase platelet-thrombus interactions, and enhance platelet adhesion and activation. Although RBCs increase blood viscosity, this effect is lessened in arteries by high shear-induced shape change. **(B)** Venous thrombi form slowly in stasis or low flow (frequently in venous valve pockets) and are RBC and fibrin rich. In veins, RBC aggregation into stacked rouleaux structures increases blood viscosity. RBCs can also directly or indirectly adhere to the vessel wall and may contribute to thrombin generation within thrombi. Once incorporated into venous thrombi, RBCs increase thrombus size and reduce thrombus permeability and susceptibility to lysis. In disease states, abnormal RBCs and RBC-derived microvesicles may also adhere to the endothelium or extracellular matrix, activate platelets and other cells, and enhance local thrombin generation during thrombosis. Adapted with permission from Byrnes and Wolberg (2).

Red blood cells (RBCs) have long been known to influence thrombosis through the visco-elastic properties of flowing blood. Moreover, it has become increasingly apparent that RBCs have multiple roles in thrombosis and hemostasis. For example, RBC deformability strongly influences clot structure and biomechanical properties, and cell to cell signaling between RBCs and endothelial cells as well as platelets contribute to both physiologic and pathologic hemostasis. RBCs also influence humoral contributions to thrombosis via RBC and RBC generated microvesicle surface phosphatidylserine interaction with the coagulation cascade. Long thought to have no active role in clot formation, RBCs are now known to be active in clot formation and contraction and to help regulate clot resolution via fibrinolysis (see Figure 1).

## Red Blood Cells and Rheology

### Viscosity

There has long been indirect evidence linking thrombosis and elevated RBC volume (Hematocrit), through effect upon blood rheological properties. In 1964, Dintenfass observed that the viscosity of blood taken from patients suffering deep venous thrombosis and coronary disease was much higher than that of healthy donors (1). Epidemiologic studies have also shown higher hematocrit to be associated with deep venous thrombosis and cardiovascular disease (2). In pathologic conditions with increased hematocrit such as cyanotic congenital heart disease and polycythemia vera, thrombosis risk is increased (3, 4). In fact, one of the treatments for polycythemia vera is hematocrit reduction through phlebotomy (4). In addition to this indirect evidence of RBC contribution to thrombosis, RBC transfusion has been reported to promote clot formation via platelet activation, particularly in the setting of thrombocytopenia (5). Historically, the relationship between RBC abundance and thrombosis was assumed to be related to viscosity; however, the contribution to viscosity by RBCs is only part of their rheologic activity.

In general, an increase in RBC abundance increases viscosity; (6) however, the viscosity-hematocrit relationship is complex. Originally, in 1935, Nygaard et al. (7) reported that the relationship between hematocrit and viscosity is non-linear; i.e., the viscosity increases in an exponential fashion above a threshold hematocrit. Initially for every 10% increase in the hematocrit, blood viscosity increases by 26% (6); whereas at higher hematocrits, a 1% increase (45–46%) increases viscosity by 4% (8). This complex relationship arises from non-Newtonian fluid properties of blood. The classification of fluids into Newtonian and non-Newtonian is based on the relationship between viscosity and shear rate (9), with Newtonian fluids demonstrating constant viscosity over differing shear rates (10) and non-Newtonian fluids having shear-dependent viscosity (9, 11). As such, in blood, RBC-viscosity relationships (and therefore hydrodynamic influence of RBCs upon clot initiation and growth) is not solely a function of the hematocrit, but is also dependent on circulatory context: shear rate, pressure gradients, vessel geometry and blood flow character (laminar vs. turbulent) (12).

### Shear Stress and Shear Rate

**Figure 2:**
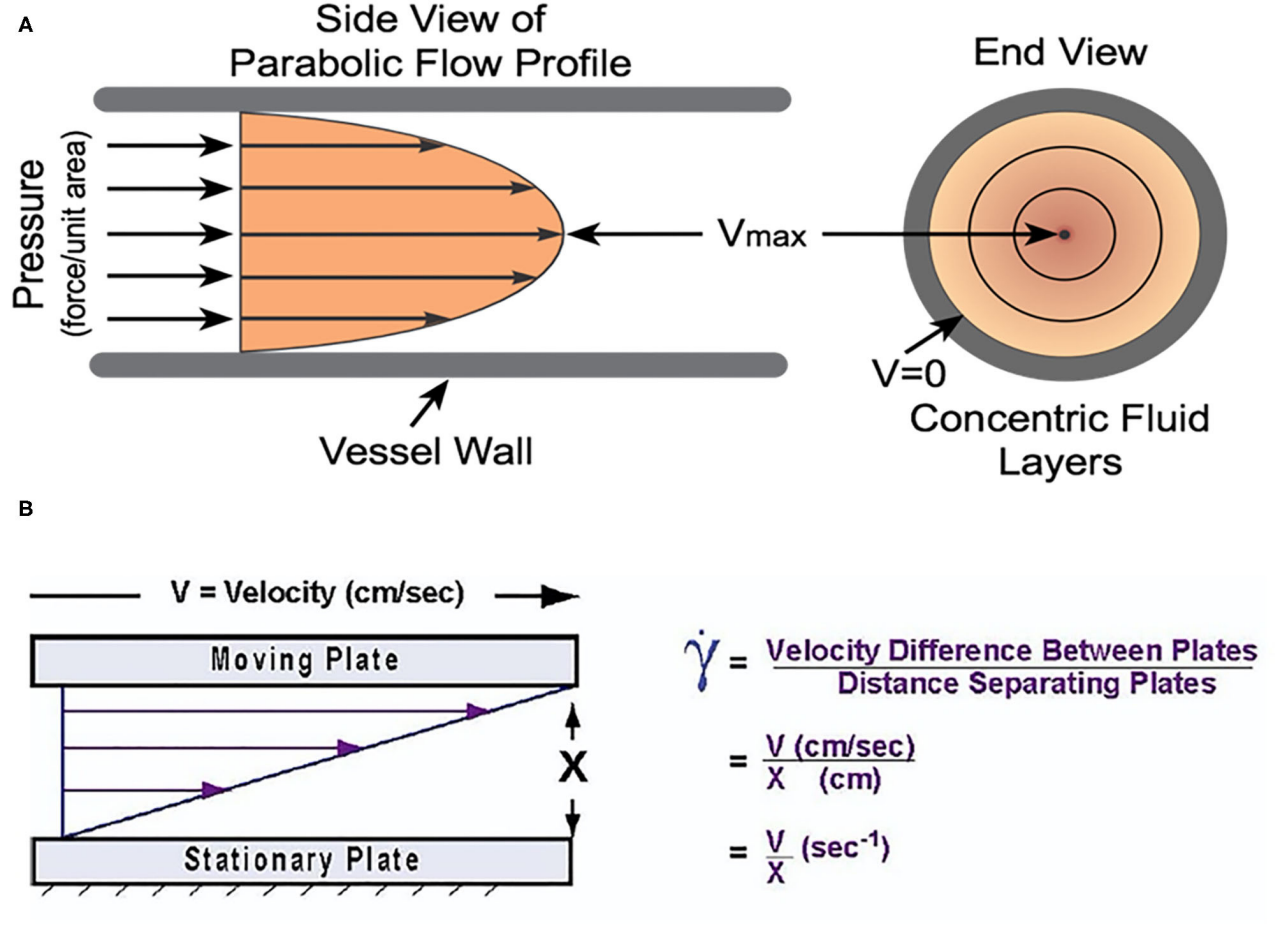
**(A)** The velocity gradient due to shear forces in blood. The fluid layers have differential velocity according to their position along the radial axis. It is worth noting that the viscosity gradient is the inverse of this gradient with the lowest viscosity at the center of the vessel and the highest nearest the vessel wall. Reprinted with permission from: Cardiovascular Physiology Concepts. cvphysiology.com Richard E. Klabunde, PhD. **(B)** The velocity gradient in blood is due to the differential forces acting upon each fluid layer due to the distance from the vessel wall. Adapted with permission from Papaioannou and Stefanadis (14).

The forces that govern RBC behavior under flow, particularly with regard to clot initiation and growth are shear stress and shear rate. Shear stress is the applied force per unit area (13), and is directly proportional to flow rate and inversely proportional to vessel diameter (14). Therefore, it is distributed in a concentric gradient within the vessel lumen—in straight segments, this gradient is inversely proportional to the third power of vessel radius (15). Shear rate is the velocity gradient between two adjacent fluid microlayers divided by their distance and also varies across the flow axis (13). *In vivo*, the instantaneous shear rate of blood not only changes along the axis of flow, but also during the cardiac cycle as a function the pulse pressure gradient (6). In a non-Newtonian fluid such as blood, viscosity is defined as the ratio of shear stress to shear rate and as such, the viscosity is lowest and velocity is highest at the vessel center; whereas, viscosity is highest and velocity is lowest at the endothelial surface. This gradient is strongly influenced by hematocrit and RBC biomechanical properties, and this physiology determines RBC aggregation (RBC-RBC association) and adhesion (RBC-endothelium association) at sites of vessel wounding or pathology—which are highly relevant for clot initiation and growth (see Figure 2).

Red cell aggregation is promoted when shear rates are low (16). RBCs assemble into stacks (rouleaux) which travel in parallel promoting plasma layering adjacent to the endothelium and preventing RBC adhesion. In addition, the RBC concentration in the vessel core concentrates platelets and coagulation factors to a more radial location in the fluid stream, which promotes interaction with endothelium. Rouleaux formation also diverts single RBCs to the flow-stream periphery and preferentially toward branching vessels. This effectively reduces the hematocrit in smaller vessels. In fact, the spatial distribution of the hematocrit throughout the circulatory tree has a coefficient of variation of 60%—as a consequence, viscosity/shear relationships (and, of course, O_2_ content) are spatially non-uniform at any given moment (17).

In smaller vessels, shear stress is higher, which prevents rouleaux formation. Not only does this decrease blood effective viscosity, but increased RBC spacing promotes interaction with the endothelial layer. RBC oxygen release velocity (related to “tank treading” and within-cell hemoglobin mixing) is also increased in smaller vessels due to higher shear stress (18), promoting oxygen release (for any given oxygen gradient) in smaller vessels and capillaries, thereby enhancing transport to metabolically active tissues. Despite the lack of rouleaux formation in small vessels, RBCs still preferentially move in the vessel center, and the more peripheral layer of cell-free plasma is maintained along endothelium (19). Even in capillary vessels (smaller diameter than RBCs), the endothelial glycocalyx (rather than the cell membrane) is the contact interface with RBCs (19). Platelets are marginated to this area which increases their concentration and potential for adhesion. However, platelets increase apparent blood viscosity more so than do red blood cells, further decreasing adjacent wall shear stress (20) and preventing shear stress induced platelet activation which is vital to maintaining vessel patency.

Endothelial cells exhibit classical mechano-transduction signaling responses to shear stress, comprising variation in protein expression and vasoactive factor release (the most notable is shear-induced nitric oxide and prostacyclin release, which link vessel tone to pulse pressure). Not only is site-specific shear stress important, but the pattern of flow informs endothelial cell signaling. The pattern of flow, i.e., laminar vs. turbulent, is mechanically transduced through the cell-free plasma layer that is immediately adjacent to endothelium (glycocalyx) (21–23). As noted above, through effect upon blood viscosity and shear properties, endothelial mechano-transduction is strongly influenced by RBC abundance (hematocrit), biomechanical (RBC deformability), and surface (aggregation and adhesion) properties. With regard to hemostatic related mechano-transduction by endothelium, the synthesis of pro-thrombotic and pro-inflammatory mediators such as Tissue Factor, von Willebrand Factor, endothelin, ICAM-1, and VCAM-1 is highly dependent on both shear stress and laminar flow (24). The expression of anticoagulant mediators such as thrombomodulin are also partially dependent on shear stress. In lower shear stress conditions where thrombosis risk is increased, thrombomodulin expression is increased; thrombomodulin binds to activated Protein C, which binds to Protein S. This protein complex inactivates factors Va and VIIIa, dampening the clotting cascade (25). In addition, platelet activation is attenuated through endothelial expression of both nitric oxide and prostacyclin (26) which also promote vessel dilation. Platelets also exhibit mechano-transduction, and are activated by high shear stress; as such vessel dilation also dampens platelet activation by lowering shear stress. Therefore, the balance between the pro- and anti-thrombotic properties of the vessels are dynamic and influenced by blood composition via the rheology-based physiology described above. Healthy vasculature adapts rapidly to the rheology-based changes within the normal dynamic range; however, in disease-related changes to rheology outside the normal dynamic range (very high or low hematocrits, or with very stiff or sticky RBCs), this compensation does not take place and alters the likelihood of both bleeding and thrombosis.

In contrast, turbulent flow (note transition thresholds for laminar vs. turbulent flow are viscosity-dependent and therefore also influenced by RBC number and character) causes endothelial cell activation, promoting RBC and leukocyte adhesion (26). Moreover, endothelium can, in fact, undergo not just biochemical, but structural changes in areas with turbulent blood flow (26). In stenotic vessels, blood flow acceleration increases the shear stress in excess of two orders of magnitude (27). Platelets are activated in areas of high shear stress (27) which then initiates the coagulation cascade. Both changes increase thrombotic risk; the relative risk for abnormal clotting consequence to vessel stenosis, therefore, is also related to RBC number and character (see Table 1).

**Table 1:** Influence of blood flow upon factors relevant to hemostasis.

**Parameter**	**Impact**
↑ laminar flow	↑ rouleaux, viscosity, and von Willebrand Factor release
↑ turbulent flow	↑ thrombomodulin, VCAM-1, and endothelin expression
↑ shear rate	↑ RBC release of O_2_, ATP and NO, platelet activation, prostacyclin release, and ICAM-1 expression

### RBC Transfusion and Venous Thromboembolism

The literature examining the influence of RBC transfusion upon hemostasis is mixed, with few formal randomized controlled trials in uniform, defined populations. Several studies in the adult literature demonstrate increased rates of bleeding after RBC transfusions in non-variceal gastrointestinal hemorrhage (28–30). However, multiple other adult studies report association between RBC transfusion and increased venous thromboembolism (VTE) risk, but these are largely retrospective and are missing key data, such as co-incident platelet and fresh frozen plasma transfusions. In the pediatric literature, an association between venous thromboembolism and RBC transfusions has also been reported; (31) similarly, these studies suffer from some of the same issues as the adult studies; a heterogenous patient population and incomplete transfusion information. Logically and in practice, the population of patients requiring RBC transfusions is inherently different than those patients who do not; designing robust analyses in this situation is therefore quite challenging.

There is, however, a plausible basis for influence of RBC transfusion upon hemostasis, with regard to the storage lesion. Moreover, as previously discussed, historical studies suggest that significant increase in viscosity alone may be sufficient to increase risk of thrombosis. Additionally, one of the main metabolic derangements that occurs with RBC storage is decreased ATP production secondary to decreased glycolysis (32). A decrease in ATP then inhibits the Na/K ATPase pump which in turn causes an influx of sodium into the RBCs with a correlative increase in size and decrease in deformability (33). The decrease in deformability can then lead to both impaired rheology as well as hemolysis which can in turn decrease nitric oxide availability secondary to scavenging from free heme. In addition to changing the size of RBCs, the loss of ATP production decreases flippase activity, increasing phosphatidylserine (PS) on the outer membrane, which along with other changes related to storage duration is associated with microvesicle (MVs) production, both of which are associated with thrombosis (34). Of note, thrombin generation is accelerated almost 3 × 10^5^ times in the presence of PS (35), leading to increased coagulation.

## Red Blood Cell Morphology

RBC morphology is integral to their function in thrombosis and hemostasis. The mechanical properties of the cytoskeleton and membrane lipid bilayer are both vital to deformation. The cytoskeleton is composed of actin filaments and spectrin tetramers (arrayed in a mesh-like fashion) that allow for deformation under stress (36). The lipid bilayer is tethered to the cytoskeleton; its composition influences fluidity and has a small role in deformability (37). The high surface to volume ratio of the red cell also is important to RBC deformability in small vessels, and changes in this ratio, such as that found in hereditary spherocytosis, determine RBC circulation time (deformability determines the success of splenic transit). Cytoplasmic viscosity and hemoglobin solubility both affect red cell membrane deformation as well (20). Increases in intracellular viscosity such as those mediated by RBC hydration and within-RBC hemoglobin concentration (e.g., the MCHC) both decrease cellular deformation by changing the hydrodynamic effective volume (37).

The RBC membrane has various components that affect cellular interaction and flow principles. For example, band-3 is the major transmembrane membrane protein and plays a role in both ion exchange and in cellular adhesion. In addition, the cytoplasmic domain of band 3 is an assembly point for other membrane proteins through binding sites that regulate the flexibility, stability, and deformability of the red cell (38). PIEZO1, a non-selective cation channel, helps to maintain red blood cell volume homeostasis (38). Defects in this channel result in hereditary xerocytosis which can lead to hemolytic anemia. PIEZO1 interacts with the Gardos channel, otherwise known as KCNN4/IK-1, which is a calcium-activated potassium channel. By governing RBC hydration, the Gardos channel helps to mediate red cell changes in shape and volume that are necessary for passage through small capillaries.

RBC deformation is important from a cell signaling perspective, a physical perspective, and from a gas and ion exchange perspective. In high shear stress situations such as near the vessel wall, RBCs adopt an elliptical shape and travel parallel to blood flow (36). This transformation prevents aggregation which lowers effective viscosity and resistance. From a physical perspective, RBCs are 7–8 microns in diameter, yet traverse 1–3 microns blood vessels (20). The normal RBC deforms to a bullet shape which maintains a high surface area near the endothelium to maximize gas exchange. Even the act of deformation promotes oxygen release (39). Red cells that are unable to deform are more likely to adhere to the vessel wall, which increases vascular resistance. Once adhered, the magnitude of the shear stress required to detach RBCs is an order of magnitude higher than that to prevent aggregation (40). Diseases characterized by abnormal RBC deformation are commonly also associated with increased risk for thrombosis. For example, in sickle cell disease, hemoglobin S polymerizes and sickled RBCs are stiffer than normal (20, 41). Not only is this important in the acute setting with vaso-occlusive crises, but over time, the rheologic properties of RBC membrane are altered leading to more rigid cells and increased thrombotic risk (41).

## Cell to Cell Signaling and Humoral Influences on Hemostasis

### Endothelium and Red Blood Cells

The endothelial glycocalyx is composed of proteoglycans, glycoproteins, and glycosaminoglycans containing heparan sulfate, chondroitin sulfate, hyaluronan, and various other proteins (22). These components are arranged in such a way as to provide both a steric and a charge dependent semipermeable barrier preventing cell adhesion (22). Therefore, under normal conditions, RBCs interact with the endothelial glycocalyx and not with the endothelial cell membrane itself *per se*. The glycocalyx also includes a high density of anticoagulant proteins such as antithrombin, heparin cofactor II, thrombomodulin, and tissue factor pathway inhibitor which prevent thrombosis in healthy vasculature (42). In pathologic conditions such as sickle cell disease and in other conditions which structurally or metabolically alter RBCs, RBC adhesion to the endothelium increases (19). Once adherent, a set of adhesion receptors is externalized that is not found on RBCs in normal conditions. The exact receptors expressed are dependent on the disease, but they are thought to overall increase the risk of thrombosis (43).

### Platelets and Erythrocytes

RBCs interact with platelets in both a mechanical and biochemical fashion. Not only do RBCs promote platelet margination through axial flow, but they also interact directly with platelets through the α_IIb_β_3_-ICAM4 receptors (43, 44). Moreover, RBCs can biochemically activate platelets through the export of ATP and ADP during high shear conditions, hypoxia, and acidosis (19, 45). Platelet aggregation is also enhanced by RBC export of thromboxane A2 (44). Platelets stimulated by RBC presence exhibit enhanced P-selectin externalization and integrin α_IIb_β_3_ activation which initiates a positive feedback loop during platelet activation (2). With RBC destruction (hemolysis), free hemoglobin molecules scavenge nitric oxide which leads to platelet disinhibition (19).

### Phosphatidylserine Exposure

The RBC membrane is a bilayer with amphiphilic molecules such as phosphatidylserine (PS) concentrated on the inner leaflet (46). Under physiologic conditions, RBC membrane proteins flippases and translocase maintain the negatively charged PS polar head orientation to the cytosolic (rather than the outer surface), which is therefore concealed from coagulation proteins (47). With high shear rates, inflammation and/or oxidative stress, flippase, and translocase are inactivated and the protein scramblase is activated (44). This change causes inversion of the PS polar head asymmetry from the inner to the outer membrane leaflet. On the outer membrane, externalized PS provides a scaffold upon which factor X can be activated to Xa and thrombin can be generated from prothrombin (46). There is a small population of senescent RBCs with PS exposure on their surfaces (~0.6%) (48). In pathologic conditions this RBC sub-population expands, and RBCs can account for up to 40% of the thrombin generation potential of whole blood (19, 49). Interestingly, however, in patients with sickle cell disease, RBC PS externalization is inversely correlated with thrombin generation (50). PS externalization also enhances RBC adherence to and activation of endothelium (43).

### Red Cell -Derived Microparticles

Membrane blebbing is common in activated, apoptotic, and aging cells. In RBCs, these processes cause both loss of membrane asymmetry via PS externalization and the release of RBC-derived microparticles (MPs), also called microvesicles (20, 51). MP release is also increased in inflammatory conditions (52). RBC-derived MPs contain five procoagulant proteins: phospholipid scramblase I, plasminogen precursor, fibrinogen beta chain precursor, complement component C9 precursor, and β2-glycoprotein I (53). MPs can activate coagulation through both the tissue factor and contact pathways of the coagulation cascade (53). Overall, increased RBC MP formation is associated with decreased clotting times (19). They are also assumed to play a role in the increased incidence of deep venous thrombosis associated with the transfusion of RBCs stored for longer duration (19). In addition to their inherent procoagulant activity, MPs are capable of scavenging and internalizing free hemoglobin, and can—through fusion—then transfer their payload to endothelium in a process that leads to vaso-occlusion in sickle cell disease (19). MPs are also released in large amounts during hemolysis; (19) therefore, they promote a pro-thrombotic milieu in conditions such as those described for microangiopathic hemolytic anemias (MAHAs) which commonly are associated with disseminated intravascular coagulation.

### Humoral Influences on Red Cells

Multiple plasma proteins found in plasma that interact with RBCs, such as thrombospondin, which is both an extracellular matrix protein and a soluble plasma protein (38) that facilitates bridging between RBCs and endothelial cells via interaction with RBCs carrying exposed phosphatidylserine. Thrombospondin is increased in multiple inflammatory conditions (54) and may play a role in pathologic thrombosis. Von Willebrand Factor is also found in plasma and facilitates endothelial cell-RBC adhesion. The mechanism of this interaction is also thought to be through phosphatidylserine and Annexin V and is shear-dependent (55). Shear stress “uncoils” and activates von Willebrand factor and increases its binding capapcity (55). ADAMTS13 is a protease that regulates the concentration of ultra-long von Willebrand factor multimers that promote a thrombotic state. There are multiple diseases in which the ADAMTS13 level or activity is low predisposing to thrombosis; (55) thrombotic thrombocytopenic purpura (TTP) is a classic example and more recently, Thrombocytopenia Associated Multiple Organ Failure (TAMOF) has been shown to also arise from diminished ADAMTS13 activity. Fibrinogen is a plasma glycoprotein whose asymmetric structure contributes to plasma viscosity and non-Newtonian behavior. It is elevated in inflammatory conditions and has been correlated with thrombotic events-even in the absence of endothelial injury (56). It is thought to be able to bind directly to RBCs, although the exact mechanism by which this occurs is yet to be fully determined. The current hypothesis is that RBCs and fibrinogen are bound through either von Willebrand factor or through an integrin receptor. High fibrinogen levels increase RBC aggregation into rouleaux which increase the local blood viscosity and promote thrombosis in low shear environments (20). Finally, immunoglobulins are found in plasma, the most important being immunoglobulin G (IgG). IgG interacts with band 3 on the red cell cytoskeleton in a non-specific fashion and is part of both physiologic processes such as the removal of aging RBCs and pathologic conditions such as autoimmune hemolytic anemia (38).

## Erythrocytes and Clot Mechanics

Long thought to be an innocent bystander of the clotting cascade, RBCs are more recently recognized as an important and active part of the thrombotic process. First, there are multiple biochemical properties of RBCs which influence the clotting cascade including interactions with platelets, von Willebrand factor, and fibrinogen. The release of RBC-derived MP's offers a surface with phosphatidylserine also is able to initiate thrombin generation in a Factor XII-dependent manner (53). Classic biochemical and flow related environments may initiate thrombosis, that is then completed with RBC incorporation into the clot structure.

Clot structure arises from a growing fibrin matrix that is stabilized by factor XIIIa crosslinking (57). RBCs are entrained in this matrix through such crosslinking. Currently it is believed that there is no bond between factor XIIIa and RBCs, but instead fibrin crosslinking secondarily traps RBCs (58). We also know that RBC presence changes the underlying fibrin structure itself in a concentration-dependent manner (59). As RBC number increases in a forming clot, the fibrin strands become larger and the clots less porous (20). In addition, RBC deformability influences elastic properties of the thrombus (59). RBC presence increases lytic resistance in the clot as well by impairing plasminogen activation and through the above-mentioned changes in fibrin structure (60). Once formed, the clot undergoes platelet-mediated contraction (20), during which entrained RBCs are compressed into shapes called polyhedrocytes, which appear between fibrin layers as “building blocks.” Finally, in a contracted clot, RBC presence modulates fibrinolytic activity both by steric inhibition and through the RBC fibrinogen receptor (59).

### Influence of Anemia Upon Bleeding Risk

Given the integral role of RBCs in hemostasis, it is important to consider how anemia may affect risk of bleeding in critically ill patients. First, although not necessarily directly correlated with the risk of bleeding, it is important to appreciate the impact of hematocrit on coagulation assay results. For example, with the PFA-100 analyzer, there is an inverse relationship between hematocrit and closure time; in fact, no platelet plug forms in this device with hematocrits <20% and, moreover, for every decrease of 50,000 in the platelet count, an increase in hematocrit by 10% will “normalize” assay output (up to hematocrits of 50%) (61). Similar findings are reported for bleeding time (anemia extends bleeding time); (62–64) however, these differences in coagulation parameters as a function of anemia do not clearly equate to altered risk of clinically significant bleeding in patients. For example, hemoglobin level is reported to independently influence bleeding risk in adults with AML (for each increase in hemoglobin of 1 g/dL, significant bleeding was reduced by 22% (RR, 0.78; 95% CI: 0.61–1); (65) however, a meta-analysis (~ 12 K subjects) evaluating restrictive v liberal RBC transfusion trials revealed a non-significant trend toward lower risk of bleeding in the restrictive (more anemic) group (66). On the other hand, in a *post-hoc* analysis of a large multicenter trial of prophylactic platelet transfusions in oncology patients (PLADO), a hematocrit ≤25 was associated with a non-significant trend toward an increased risk of clinically significant bleeding (OR 1.29; 95%CI: 0.98–1.47); however, there was no observed interaction between RBC transfusion and next-day bleeding, as a function of anemia severity (67). Of note, the above CI barely dips below 1 and as in many situations, it may be reasonable to consider the difference between statistically significant and clinically significant information. As may be apparent from other material in this paper, it is probable that there is a threshold below which anemia is a clinically relevant risk factor for bleeding, likely in synergy with abnormalities in hemostasis, itself (such as low platelets, fibrinogen, etc.). This issue is reviewed thoughtfully in an editorial accompanying the *post-hoc* PLADO analysis (68). Therefore, at this time, while there is no clear empiric support for targeting a specific hemoglobin level to prevent bleeding complications; in patients with clinically significant bleeding that is refractory to appropriate correction of coagulopathy, or in patients with uncorrectable defects in hemostasis (persistent thrombocytopenia or hypofibrinogenemia), there may be plausible biologic justification to target a higher hematocrit goal than would be pursued under other circumstances.

## Conclusion

Overall, RBCs have a significant impact on the hemostatic process, both actively and passively. Through multiple facets including rheology, morphology, cell signaling and humoral interactions, and physical presence in blood clots, RBCs contribute to hemostasis. Appreciation of the extent of this contribution will likely continue to expand in the future and may possibly offer novel therapeutic targets for modulating hemostasis (see Table 2).

**Table 2:** Summary of modalities through which RBCs contribute to hemostasis.

• Effect upon humoral and endothelial mechano-signaling *via* altered blood flow
• RBC membrane surface as physical interface with other hemostatic elements
• RBC membrane surface as biochemical/signaling trigger and accelerant in coagulation cascade
• Direct signaling *via* exported biomolecules from RBCs to endothelial cells and platelets
• Elaboration of RBC microparticles, with both mechanical and signaling effects
• Contribution of intact RBCs to 3D geometry and biophysical properties of thrombi

## Author Contributions

AG researched the current literature and completed the first draft of the article. AD oversaw the writing of the article, discussed the relevant research, and edited the draft. All authors contributed to the article and approved the submitted version.

## Conflict of Interest

The authors declare that the research was conducted in the absence of any commercial or financial relationships that could be construed as a potential conflict of interest.
